# Immunogenicity and Effectiveness of Routine Immunization With 1 or 2 Doses of Inactivated Poliovirus Vaccine: Systematic Review and Meta-analysis

**DOI:** 10.1093/infdis/jit601

**Published:** 2014-11-01

**Authors:** Nicholas C. Grassly

**Affiliations:** Department of Infectious Disease Epidemiology, St Mary's Hospital Medical School, Imperial College London, United Kingdom

**Keywords:** inactivated poliovirus vaccine, IPV, poliomyelitis, immunogenicity, seroconversion, efficacy, eradication, endgame, antibody

## Abstract

***Background.*** The World Health Organization has recommended that all 124 countries currently using only oral poliovirus vaccine (OPV) introduce at least 1 dose of inactivated poliovirus vaccine (IPV) before the global withdrawal of serotype 2 OPV in 2016. A 1- or 2-dose schedule, potentially administered intradermally with reduced antigen content, may make this affordable.

***Methods.*** A systematic review and meta-analysis of studies documenting seroconversion after 1 or 2, full or fractional (1/5) doses of enhanced-potency IPV was performed. Studies reporting the clinical efficacy of IPV were also reviewed.

***Results.*** Twenty study arms from 12 published articles were included in the analysis of seroconversion. One full dose of intramuscular IPV seroconverted 33%, 41%, and 47% of infants against serotypes 1, 2, and 3 on average, whereas 2 full doses seroconverted 79%, 80%, and 90%, respectively. Seroconversion increased with age at administration. Limited data from case-control studies indicate clinical efficacy equivalent to the proportion seroconverting. One fractional dose of intradermal IPV gave lower seroconversion (10%–40%), but after 2 doses seroconversion was comparable to that with full-dose IPV.

***Conclusions.*** Routine immunization with 2 full or fractional doses of IPV given after 10 weeks of age is likely to protect >80% of recipients against poliomyelitis if poliovirus reemerges after withdrawal of OPV serotypes.

The Global Polio Eradication Initiative (GPEI) Strategic Plan for 2013–2018 envisages globally coordinated withdrawal of the live oral poliovirus vaccine (OPV), based on the long-recognized need to prevent the emergence and spread of vaccine-derived polioviruses [1, 2]. Withdrawal will happen serotype by serotype, starting with serotype 2 OPV (OPV2) by mid-2016, given the global elimination of this serotype of wild poliovirus (the last naturally occurring case was reported in India in 1999). Routine immunization programs will therefore replace trivalent OPV with serotype 1 and 3 bivalent OPV, and serotype 2–containing OPV will no longer be used during supplementary immunization activities. Subsequent withdrawal of serotypes 1 and 3 will follow, contingent on the certified elimination of transmission of these serotypes, such that OPV will no longer be used as of 2019.

Global withdrawal of OPV will put the 124 countries currently using a trivalent OPV schedule at risk of outbreaks of poliomyelitis should poliovirus be reintroduced. In November 2012, the World Health Organization Strategic Advisory Group of Experts (SAGE) on Immunization therefore recommended that “all countries should introduce at least 1 dose of IPV in their routine immunization schedules to mitigate the risks associated with the withdrawal of OPV2” [3]. Individuals vaccinated with IPV would be protected against poliomyelitis in the event of an outbreak of vaccine-derived or wild-type poliovirus. A degree of herd immunity would also be provided by this vaccine, thereby limiting transmission of any reintroduced or reemergent poliovirus. Following withdrawal of OPV2, but before complete OPV cessation, IPV may provide additional benefits in terms of boosting immunity to serotypes 1 and 3, which could help wild poliovirus eradication, and preventing vaccine-associated paralytic poliomyelitis (VAPP) in any seronegative individuals subsequently vaccinated with bivalent or monovalent OPV.

A prerequisite for the universal adoption of IPV in routine immunization is the availability of an affordable product [3]. The higher cost of manufacture and limited market volume has meant that the price of stand-alone IPV is at least 10 times that of OPV. Even with a larger market volume, the price of stand-alone IPV is unlikely to drop much below US $1 per dose [4]. For this reason, the GPEI is supporting multiple strategies for reduced-cost IPV, including evaluation of 1- or 2-dose schedules, licensing of products with a reduced antigen content based on intradermal delivery or the use of adjuvants, and pursuit of cheaper production costs by using safer vaccine seed strains including Sabin or further attenuated polioviruses (IPV manufacture is traditionally based on inactivation of wild-type strains). Over the timeframe envisaged for serotype 2 OPV withdrawal, currently licensed stand-alone IPV is likely to meet most of the demand from countries currently using trivalent OPV. The budget for the GPEI Strategic Plan therefore includes funds to support the universal introduction of 1 dose of IPV to routine immunization schedules. Intradermal delivery of a fractional (1/5) dose may also be an option if regulatory approval can be obtained for a label change to include intradermal delivery from a multidose vial. A reduced-antigen-content, alum-adjuvanted product may also be achievable in the medium term depending on licensing and regulatory approval [1]. Multivalent vaccines containing IPV may be an option for some countries, and in the longer term these vaccines may meet demand for polio immunization in middle and low-income countries. However, current pricing of these products, limited production capacity, the absence of a hexavalent product containing whole cell pertussis, and the need for 3 doses means that these products are unlikely to be an option in the medium term [4].

In the short term, countries currently using trivalent OPV will need to make a decision on the most appropriate IPV schedule and product. The evidence base for this decision is limited in the case of 1- or 2-dose schedules, as most studies have focused on immunization with at least 3 doses of IPV. A single dose of IPV can prime individuals for a subsequent booster in the event of an outbreak. It may also protect against poliomyelitis and poliovirus transmission, although the relationship between priming and vaccine efficacy is not well understood [5]. In this article, I review the immunogenicity and effectiveness of 1- and 2-dose IPV schedules given as a stand-alone or combination product, at full or reduced antigen content via the intramuscular or intradermal route to healthy children. I do not consider >2-dose schedules, as there is an ample evidence base and experience with their use [6, 7]. This article is therefore intended as a resource to decision makers considering the different policy options for the introduction of IPV to routine immunization before the global withdrawal of OPV serotypes that is planned to commence in 2016.

## SEROCONVERSION DATA

Studies reporting seroconversion after 1 or 2 full or fractional (1/5) doses of IPV were identified through a systematic review of published studies. The protocol for this review is described in the Supplementary Methods. A total of 20 independent study arms were identified in 12 published articles after screening 958 articles returned by a search of the Web of Knowledge collection of databases (Supplementary Figure 1).

### Single Dose

**Figure 1.**
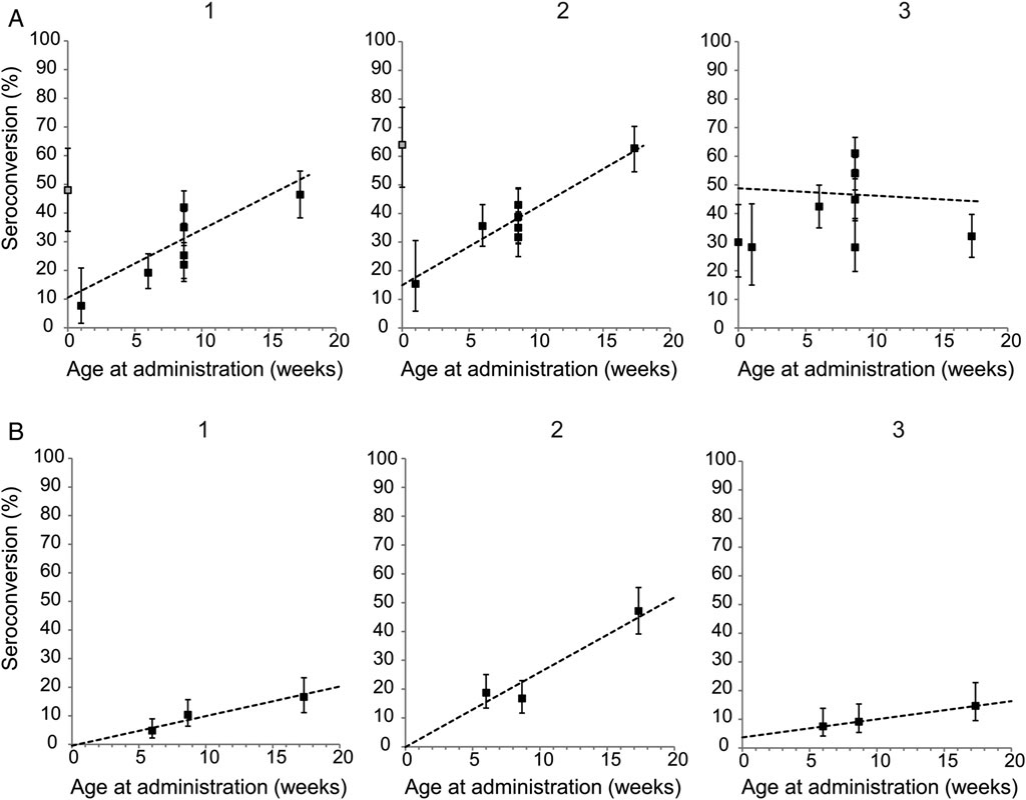
Proportion of children seroconverting to each serotype after 1 dose of inactivated poliovirus vaccine (IPV), plotted against age at administration. *A*, Full-potency IPV given intramuscularly. *B*, Fractional (1/5) dose IPV given intradermally using a needle-free device. The dashed line shows the maximum-likelihood linear trend (likelihood ratio test for trend *P* <.001, <.001, and .49 for serotypes 1, 2, and 3 in *A*, and *P* <.001, <.001, and .027, respectively, in *B*). The error bars show the 95% confidence intervals for the data. Seroconversion was defined in each study as either a 4-fold rise in neutralizing antibody titer compared with that expected based on the expected decline in maternal antibodies, or detection of neutralizing antibodies in children previously seronegative (see Supplementary Table 1 for details for each study). Seroconversion data from a study of IPV given at birth in India is shown with a gray fill for serotypes 1 and 2 (*A*), as a high proportion of infants showed seroconversion to these serotypes in the control arm of the study where IPV was not administered at birth [8]. Data from this study for serotypes 1 and 2 were therefore not included when estimating the best-fit linear trend.

Seven studies were identified that reported seroconversion after a single dose of enhanced potency IPV given by intramuscular injection. These 7 studies included results from a total of 8 independent study arms (Supplementary Table 1). The overall proportion of children seroconverting after a single dose was 33%, 41%, and 47% for serotypes 1, 2, and 3 respectively, although there was significant heterogeneity among studies (χ^2^ test for heterogeneity, *P* < .001 for all 3 serotypes). In particular, the proportion seroconverting was strongly dependent on the age at administration for serotypes 1 and 2, ranging from 8%–15% when given 1 week after birth, to 46%–63% when given at 4 months of age (Figure 1). This trend was less apparent for serotype 3, with a nonsignificant, negative linear trend with age at administration, although this was because of a single study with poor serotype 3 immunogenicity of IPV given at 4 months of age [9].

Three studies reported seroconversion after a single fractional (1/5) dose of enhanced potency IPV administered via the intradermal route, all using a needle-free device (Biojector 2000, Bioject Medical Technologies). The overall proportion of children showing seroconversion was 10%, 27%, and 10% for serotypes 1, 2, and 3 respectively, significantly lower than that observed after the full-dose product administered intramuscularly (mixed-effects binomial regression *P* < .001 for all 3 serotypes; Figure 1, Supplementary Table 1). In all 3 studies the geometric mean titer (GMT) of poliovirus serum neutralizing antibodies was lower after a single fractional dose of intradermal IPV compared with children who received a full dose intramuscularly [9–11].

### Two Doses

**Figure 2.**
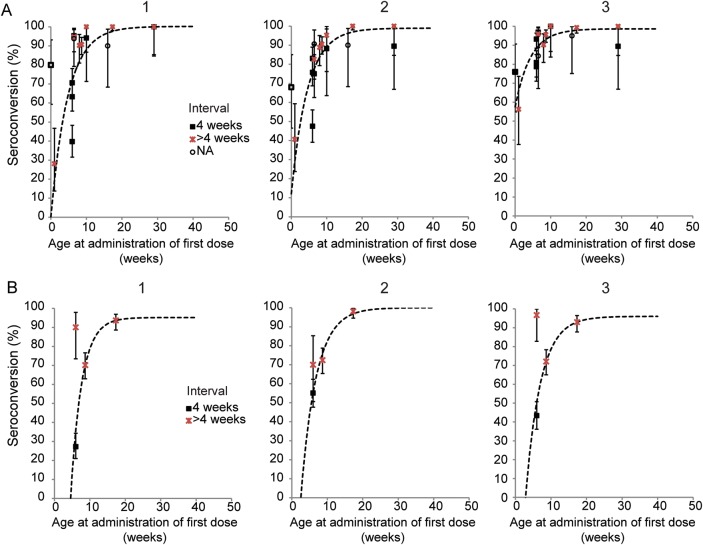
Proportion of children seroconverting to each serotype after 2 doses of inactivated poliovirus vaccine (IPV), plotted against age at administration of the first dose. *A*, Full-potency IPV given intramuscularly. *B*, Fractional (1/5) dose IPV given intradermally using a needle and syringe or needle-free device. The legend indicates the interval between the doses, and the error bars the 95% confidence intervals. The dashed line indicates the trend in seroconversion with age at administration of the first dose based on an exponential curve with intercept and asymptote estimated from the data using maximum likelihood. The asymptote was constrained to be ≤100%. Seroconversion is defined as in Figure 1 (see Supplementary Table 2 for details for each study). Data from the study in India with significant seroconversion to serotypes 1 and 2 in the control arm are shown in gray as for Figure 1 and excluded from the maximum likelihood curve fit [8].

Ten articles were identified that reported seroconversion after 2 doses of enhanced-potency IPV given by intramuscular injection in a total of 16 independent study arms. The overall proportion of children seroconverting after 2 doses was 79%, 80%, and 90% for serotypes 1, 2, and 3 respectively, with significant heterogeneity among studies (χ^2^ test for heterogeneity, *P* < .001 for all 3 serotypes; Supplementary Table 2). Seroconversion increased with age at which the first dose was administered, with most studies showing at least 80% seroconversion for each serotype when the first dose was given at 10 weeks after birth or later (Figure 2). An interval of 4 weeks between the first and second dose was associated with a lower proportion of children seroconverting compared with longer intervals between doses (average seroconversion of 65%, 71%, and 87% for 4-week interval compared with 90%, 89%, and 93% for a longer interval—on average 9 weeks—for serotypes 1, 2, and 3 respectively, although this was nonsignificant based on mixed-effects binomial regression, *P* = .56, .084, and .59 for serotypes 1, 2, and 3, respectively).

Four studies reported seroconversion after 2 fractional (1/5) doses of enhanced potency IPV administered via the intradermal route using either a needle and syringe or needle-free device. The proportion of children showing seroconversion was 63%, 74%, and 70% on average for serotypes 1, 2, and 3, respectively, which was significantly lower than that observed after the full-dose product was administered intramuscularly (mixed-effects binomial regression, *P* < .001 for all 3 serotypes; Supplementary Table 2). However, even for this limited sample size, seroconversion appeared dependent on the age at administration of the first dose and the interval between the doses (Figure 2). Excluding the single study that administered a fractional dose of IPV at 6 and 10 weeks [11] increased the average seroconversion to 82%, 83%, and 83% for serotypes 1, 2, and 3, respectively. Nonetheless, after 2 doses the GMT of poliovirus-specific serum neutralizing antibodies remained lower among children receiving fractional-dose intradermal IPV compared with full-dose product intramuscularly in all studies that directly compared these products—and this difference persisted even after 3 doses [9–11].

## PRIMING

Serum neutralizing antibodies are thought to be the major determinant of protection against poliomyelitis, although cellular immunity also plays a role [12]. The protective effect of antibody was determined during early attempts to prevent poliomyelitis through the administration of gamma globulin [13]. Seroconversion is therefore the standard measure of protective immunity against poliomyelitis following immunization with IPV. However, it has also been argued that even in the absence of detectable antibody, immunological memory following administration of IPV may be sufficient to protect against poliomyelitis [5, 14].

Recent data from Cuba suggest that immunological priming develops in the majority (at least 90%) of children following immunization with a single full intramuscular or fractional (1/5) intradermal dose of IPV given at 4 months of age, despite more limited seroconversion (range, 17%–63% depending on serotype and route of administration; [9]). In this study, priming was evidenced by rapid seroconversion, 7 days after administration of a subsequent dose of IPV at 8 months of age. A similar anamnestic response was documented in at least 80% of infants immunized with full-dose IPV at birth, when given a booster dose at 6 months of age or older [15]. Earlier studies also provided evidence for priming in the majority of children when immunized with different IPV preparations at 5 months of age [16].

## IMMUNE MEMORY

After 3 or 4 doses of IPV, including a booster dose in the second year of life, serum neutralizing antibodies to poliovirus remain at a detectable level in the majority of recipients for many years [6]. The titer of neutralizing antibody drops quite steeply—by approximately 10- to 100-fold—in the first 2 years after vaccination, but then declines more slowly in the subsequent decade [17, 18]. Even in the absence of detectable antibodies, an anamnestic response to a booster dose can be observed at least 8 years after a primary immunization series with IPV [19]. Furthermore, in elderly individuals born before routine immunization against poliomyelitis, similar memory responses are frequently observed following challenge with IPV or OPV despite the absence of serum neutralizing antibodies, presumably because of historic exposure to circulating poliovirus [20, 21].

The extent of poliovirus neutralizing antibody decline following immunization with IPV has been shown to depend on the degree of the response to the primary immunization series. Administration of smaller quantities of antigen during primary intramuscular immunization is associated with both a lower initial titer of neutralizing antibody and reduced persistence of detectable antibody [22, 23]. Furthermore, the lower titer of neutralizing antibody in individuals immunized with fractional doses remains apparent even after a booster dose with higher antigen content [22]. However, there is no experience with the long-term persistence of immunity following intradermal administration of fractional doses of IPV. The GMT following immunization with 1–3 doses of fractional (1/5) dose intradermal IPV is generally lower than that following full-dose intramuscular IPV and so it might be expected that titers would drop below the threshold for detection earlier [9–11]. However, whether this translates into lower protection against paralytic poliomyelitis is unknown and will depend on the degree of protection offered by residual immune memory.

The duration of the primed state following a single dose of intramuscular or intradermal IPV is also unknown. Anamnestic responses following a booster dose have been observed in studies up to 12 months after an initial single dose of IPV [15, 24]. Indeed, the extent of the antibody rise after boosting appears to increase with an increasing interval between the priming and booster dose up to about 6 months [24]. However, whether the primed state following a single dose of IPV persists beyond 12 months in the absence of immune boosting is unknown.

## PROTECTION AGAINST POLIOMYELITIS

The development of serum neutralizing antibodies detectable at a dilution of 1 in 8 or higher is typically taken as a marker of protective immunity against poliomyelitis following vaccination with IPV [6]. Immunological memory developed after a primary immunization series of 3–5 doses of IPV is also likely to be protective even in the absence of detectable neutralizing antibody in serum. The epidemiological observation of an absence of poliomyelitis in countries with long-term routine use of IPV supports this belief. Nonetheless, in some countries an adult booster with IPV is recommended for travelers to regions with continued poliovirus circulation.

It is less clear whether immunological priming after a single dose of IPV is protective against paralytic disease. Jonas Salk, the developer of IPV, believed that immunological memory following a single dose of IPV was protective against paralysis [5]. Analyzing poliovirus serology data from paralyzed and healthy children before the introduction of vaccines, he noted that children with antibodies only to serotype 2 appeared to be less likely to become paralyzed by serotype 1 compared with naive children [25]. He argued that this apparent heterotypic protection was analogous to homotypic priming by a single dose of IPV in the absence of homotypic antibody. However, there are limited data to support this argument, and Salk accepted that 2 doses of vaccine would be prudent when the first dose needs to be given before 6 months of age [5].

In Hungary, campaigns with monovalent OPVs as a method of poliomyelitis prevention were replaced in 1992 by routine immunization with a single dose of enhanced-potency IPV given at 3 months of age followed by 5 doses of trivalent OPV [26, 27]. In 2006 this sequential schedule was replaced with one based on IPV alone. No cases of VAPP were reported during 1992–2006, compared with 54 cases between 1961 and 1990 when only monovalent OPVs were in use [27]. This suggests that a single dose of IPV provides some protection against poliomyelitis. However, challenge with attenuated poliovirus, albeit at a high titer, is clearly different from exposure to the highly adapted wild-type poliovirus.

There are limited epidemiological data on the protective efficacy of a single dose of enhanced potency IPV. In Senegal, a case-control analysis during an outbreak of poliomyelitis in 1986–1987 resulted in an estimated efficacy of 36% after a single dose, although with very broad 95% confidence intervals (CIs) of 0%–67% [28]. The efficacy of 2 doses in this same study was 89% (95% CI, 62%–97%). In a pilot study of enhanced potency IPV in the North Arcot district of southern India, the protective efficacy of a single dose was estimated at 52% (95% CI, 10%–75%) and of 2 doses at 67% (95% CI, 37%–83%) using a case-control method applied to 210 cases reported during 1989–1992 (V. Balraj, R. Samuel, T. J. John, A. Hall, personal communication [3 June 2013]). The effectiveness of a single dose of first-generation IPV was inferred by Salk from the distribution of the number of individuals with poliomyelitis reported to have received 0 or 1 doses during 1959 in the United States [23]. Although this case-control analysis did not attempt to control for any confounders, the protective efficacy of a single dose was 44% and of 2 doses was 82%, similar to that observed in Senegal and India. These estimates are also quite similar to the observed proportion of children seroconverting after a single dose of IPV (Figure 1). However, they should be interpreted with some caution—it is difficult to control for differences in exposure between cases and controls, particularly where multiple serotypes may be circulating, and this can lead to underestimation of the degree of protection offered by vaccination. Nonetheless, it is clear that a single dose of IPV is less effective than 2 doses against poliomyelitis.

There are no comparable studies with fractional-dose intradermal IPV because this vaccine has not been used in large-scale immunization studies.

## DISCUSSION

A single, full dose of IPV administered intramuscularly at 3–4 months of age (corresponding to the second or third diphtheria-pertussis-tetanus [DPT] visit in many countries) will seroconvert approximately 50% of recipients. The limited data available suggest the protective efficacy of this vaccine against poliomyelitis would also be about 50%. The majority of recipients would also be immunologically primed (probably at least 90%), and they would therefore respond rapidly to a subsequent dose of vaccine. Earlier administration of vaccine results in poorer seroconversion as a result of interference by maternal antibodies [10, 11]. Coverage with the third dose of DPT is somewhat lower than with the first dose, and this may be marked in countries with poor overall routine immunization coverage. Furthermore, vaccination is typically delayed—by a median of 2 and 6 weeks for the first and third dose of DPT, respectively, in low- and middle-income countries [29, 30]. Therefore, if possible, administration of IPV should occur at the most appropriately timed visit, rather than be linked to delivery of a specific dose of DPT. Seroconversion may be higher among children receiving the vaccine at older ages, but no studies examined seroconversion after 1 dose beyond 4 months of age. If VAPP prevention while bivalent OPV continues to be used was of concern, then IPV could be given earlier [31].

Seroconversion is more limited after a single fractional (1/5) dose of IPV administered intradermally, with approximately 10%–40% expected to seroconvert when this vaccine is given at 3–4 months of age. There is no experience with the clinical efficacy of a single fractional-dose IPV, although it is also likely to be lower than for full-dose product.

Two full doses of IPV will seroconvert at least 80% of children when the first dose is administered at 10 weeks of age or later. A similar proportion of children will be protected against poliomyelitis based on available efficacy data. Seroconversion is better if the 2 doses are administered >4 weeks apart, although in the one identified study in which the first dose was given at approximately 10 weeks and the second 4 weeks later, seroconversion occurred in 94%, 88%, and 100% of 17 infants [32]. These data therefore suggest that full-dose IPV given at the second and third DPT visit would be sufficiently immunogenic to protect at least 80% of recipients from poliomyelitis, although immunogenicity will be better where DPT is given later than the 6-, 10-, and 14-week schedule implemented in many countries (this is also apparent after 3 doses of IPV [33]). Alternatively, the first IPV dose could be given at the third DPT visit and the second with measles vaccine at 9 months or thereabouts. This may be favorable because the duration of protection offered by 2 doses of IPV has not been established, although it is known that persistence of detectable serum neutralizing antibodies to poliovirus is dependent on the strength of response to the initial immunization series [22, 23]. A longer interval between the first and second dose would result in higher antibody titers and therefore potentially a more durable response [24]. However, studies to formally establish the immunogenicity of this schedule are required. The level of coverage achieved with a measles vaccine given at 9 months should also be taken into account when considering this option.

Only one identified study examined the immunogenicity of 2 fractional (1/5) doses of enhanced potency intradermal IPV when the first dose was given after 10 weeks of age. This study showed seroconversion in 94%, 98%, and 93% of infants receiving fractional-dose intradermal IPV administered with a needle-free device at 4 and 8 months for serotypes 1, 2, and 3, respectively, compared with 100%, 100%, and 99% of infants receiving the full-dose product intramuscularly [9]. In general, the seroconversion data suggest that comparable protection against poliomyelitis would be offered by 2 doses of fractional-dose intradermal IPV compared with that achieved with full-dose product (approximately 80%; Figure 2). A note of caution is required, however, as neutralizing antibody GMT was consistently lower after fractional-dose product and the clinical efficacy of this vaccine has not been evaluated.

There are a number of limitations with the studies of seroconversion included in the systematic review. First, in the presence of maternal antibodies, seroconversion in the infant must be defined with reference to the expected antibody titer at the time of sample collection in the absence of vaccination. This is typically inferred from the titer measured at baseline and a half-life of approximately 28–30 days. It is possible that this method will over- or underestimate seroconversion if the decline in antibody titer deviates from this pattern. Second, children may seroconvert as a result of exposure to wild poliovirus or secondary spread of vaccine poliovirus by children vaccinated with OPV. The former is relatively rare, although it was clearly apparent in one study included in the systematic review [8]. Secondary exposure to vaccine poliovirus could have occurred in all studies based on their date and location, with the exception of the 2 Cuban studies that were timed to occur between national immunization days with OPV [9, 11]. The proportion of children seroconverting in these 2 studies was consistent with the results from the other studies after adjusting for the age at administration (Supplementary Tables 1 and 2). Finally, study staff could not be blinded to the vaccine administered when fractional intradermal and full-dose intramuscular IPV were compared. However, laboratory testing was blind to the vaccine administered in at least 3 of the 4 studies, minimizing the risk of any potential bias.

IPV clearly induces herd immunity sufficient to eliminate poliovirus transmission in high-income countries [34]. However, there is very limited experience of the impact of this vaccine on poliovirus transmission in middle- and low-income countries. Mucosal immunity induced by IPV against poliovirus shedding in stool is significantly reduced compared with OPV [35]. The impact of this vaccine in countries where poliovirus transmission is more efficient could also be limited, particularly if the fecal-oral route is important. Vaccination coverage achieved by routine immunization programs in these settings should also be considered when estimating the likely impact of IPV on transmission of any reintroduced or reemergent poliovirus. A recent mathematical modeling study suggested that routine immunization with IPV would be beneficial under most outbreak scenarios, although rarely it could allow “silent” transmission by preventing poliomyelitis but not poliovirus shedding [36]. There was considerable uncertainty about the degree of benefit, given limited data on the impact of IPV on poliovirus transmission in different settings.

Irrespective of the degree of herd immunity provided by IPV, it can clearly mitigate the health impact of any reintroduced or reemergent poliovirus after the global withdrawal of OPV serotypes, by protecting individuals from paralytic disease. A single full dose of IPV given at 3–4 months of age is likely to protect about half of recipients, while 2 full doses administered after 10 weeks of age would protect at least 80%. Therefore, in countries at risk of poliovirus outbreaks, 2 doses are likely to be preferred over a single dose. Fractional dosing may make this option more affordable, and seroconversion after 2 doses was comparable with the full-dose product (but not after 1 dose). However, there is no programmatic experience with this vaccine, and its effectiveness will depend on the feasibility of intradermal administration with needles or needle-free devices and the associated costs.

## Supplementary Data

Supplementary materials are available at *The Journal of Infectious Diseases* online (http://jid.oxfordjournals.org/). Supplementary materials consist of data provided by the author that are published to benefit the reader. The posted materials are not copyedited. The contents of all supplementary data are the sole responsibility of the authors. Questions or messages regarding errors should be addressed to the author.

Supplementary Data

## References

[1] Global Polio Eradication Initiative Polio eradication and endgame strategic plan 2013–2018.

[2] World Health Organization (2004). Conclusions and recommendations of the Ad Hoc Advisory Committee on Poliomyelitis Eradication, Geneva, 21–22 September 2004. Wkly Epidemiol Rec.

[3] World Health Organization (2013). Meeting of the Strategic Advisory Group of Experts on immunization, November 2012—conclusions and recommendations. Wkly Epidemiol Rec.

[4] Oliver Wyman Inc (2009). Global post-eradication IPV supply and demand assessment: integrated findings. Commissioned by the Bill & Melinda Gates Foundation.

[5] Salk J (1984). One-dose immunization against paralytic poliomyelitis using a noninfectious vaccine. Rev Infect Dis.

[6] Plotkin AA, Vidor E, Plotkin SA, Orenstein WA (2004). Poliovirus vaccine—inactivated. Vaccines.

[7] Vidor E, Meschievitz C, Plotkin S (1997). Fifteen years of experience with Vero-produced enhanced potency inactivated poliovirus vaccine. Pediatr Infect Dis J.

[8] Jain PK, Dutta AK, Nangia S, Khare S, Saili A (1997). Seroconversion following killed polio vaccine in neonates. Indian J Pediatr.

[9] Resik S, Tejeda A, Sutter RW (2013). Priming after a fractional dose of inactivated poliovirus vaccine. New Engl J Med.

[10] Mohammed AJ, AlAwaidy S, Bawikar S (2010). Fractional doses of inactivated poliovirus vaccine in Oman. N Engl J Med.

[11] Resik S, Tejeda A, Lago PM (2010). Randomized controlled clinical trial of fractional doses of inactivated poliovirus vaccine administered intradermally by needle-free device in Cuba. J Infect Dis.

[12] Wahid R, Cannon MJ, Chow M (2005). Virus-specific CD4+ and CD8+ cytotoxic T-cell responses and long-term T-cell memory in individuals vaccinated against polio. J Virol.

[13] Hammon WM, Coriell LL, Ludwig EH (1954). Evaluation of Red Cross gamma globulin as a prophylactic agent for poliomyelitis. 5. Reanalysis of results based on laboratory-confirmed cases. J Am Med Assoc.

[14] Salk D, Vanwezel AL, Salk J (1984). Induction of long-term immunity to paralytic poliomyelitis by use of non-infectious vaccine. Lancet.

[15] Swartz TA, Handsher R, Stoeckel P (1989). Immunologic memory induced at birth by immunization with inactivated polio vaccine in a reduced schedule. Eur J Epidemiol.

[16] Salk J, Stoeckel P, Vanwezel AL, Lapinleimu K, Vansteenis G (1982). Antigen content of inactivated poliovirus vaccine for use in a one-dose or 2-dose regimen. Ann Clin Res.

[17] Bottiger M (1990). Polio immunity to killed vaccine—an 18-year follow-up. Vaccine.

[18] Faden H, Duffy L, Sun M, Shuff C (1993). Long-term immunity to poliovirus in children immunized with live attenuated and enhanced-potency inactivated trivalent poliovirus vaccines. J Infect Dis.

[19] Bottiger M (1973). Antibody stimulation in individuals without demonstrable poliovirus antibodies following a fifth injection of inactivated poliovirus vaccine. Acta Path Micro Im B.

[20] Rumke HC, Oostvogel PM, Vansteenis G, Vanloon AM (1995). Poliomyelitis in the Netherlands—a review of population immunity and exposure between the epidemics in 1978 and 1992. Epidemiol Infect.

[21] Abbink F, Buisman AM, Doornbos G, Woldman J, Kimman TG, Conyn-van Spaendonck MA (2005). Poliovirus-specific memory immunity in seronegative elderly people does not protect against virus excretion. J Infect Dis.

[22] Salk JE (1958). How many injections of poliomyelitis vaccine for effective and durable immunity. J Am Med Assoc.

[23] Salk JE (1960). Persistence of immunity after administration of formalin-treated poliovirus vaccine. Lancet.

[24] Salk J (1982). Future prospects for vaccination against virus diseases. Behring Institute Mitteilungen.

[25] Salk JE (1956). Requirements for persistent immunity to poliomyelitis. Trans Assoc Am Physicians.

[26] Estivariz CF, Molnar Z, Venczel L (2011). Paralytic poliomyelitis associated with Sabin monovalent and bivalent oral polio vaccines in Hungary. Am J Epidemiol.

[27] Kapusinszky B, Molnar Z, Szomor KN, Berencsi G (2010). Molecular characterization of poliovirus isolates from children who contracted vaccine-associated paralytic poliomyelitis (VAPP) following administration of monovalent type 3 oral poliovirus vaccine in the 1960s in Hungary. FEMS Immunol Med Microbiol.

[28] Robertson SE, Drucker JA, Fabreteste B (1988). Clinical efficacy of a new, enhanced-potency, inactivated poliovirus vaccine. Lancet.

[29] Clark A, Sanderson C (2009). Timing of children's vaccinations in 45 low-income and middle-income countries: an analysis of survey data. Lancet.

[30] Akmatov MK, Mikolajczyk RT (2012). Timeliness of childhood vaccinations in 31 low and middle-income countries. J Epidemiol Community Health.

[31] Miller MA, Sutter RW, Strebel PM, Hadler SC (1996). Cost-effectiveness of incorporating inactivated poliovirus vaccine into the routine childhood immunization schedule. J Am Med Assoc.

[32] Simoes EA, Padmini B, Steinhoff MC, Jadhav M, John TJ (1985). Antibody-response of infants to 2 doses of inactivated poliovirus vaccine of enhanced potency. Am J Dis Child.

[33] Dayan GH, Thorley M, Yamamura Y (2007). Serologic response to inactivated poliovirus vaccine: a randomized clinical trial comparing 2 vaccination schedules in Puerto Rico. J Infect Dis.

[34] Murdin AD, Barreto L, Plotkin S (1996). Inactivated poliovirus vaccine: past and present experience. Vaccine.

[35] Hird TR, Grassly NC (2012). Systematic review of mucosal immunity induced by oral and inactivated poliovirus vaccines against virus shedding following oral poliovirus challenge. PLoS Pathog.

[36] Mangal TD, Aylward RB, Grassly NC (2013). The potential impact of routine immunization with inactivated poliovirus vaccine on wild-type or vaccine-derived poliovirus outbreaks in a posteradication setting. Am J Epidemiol.

