# Supplementary material for: Immunogenicity and Effectiveness of Routine Immunization With 1 or 2 Doses of Inactivated Poliovirus Vaccine: Systematic Review and Meta-analysis
Source: J Infect Dis. 2014 Nov 1;210(Suppl 1):S439–46. doi: 10.1093/infdis/jit601 (PMC4197908; doi:10.1093/infdis/jit601)
Supplement: Supplementary Data [file supp_210_suppl-1_S439__index.html]

Immunogenicity and Effectiveness of Routine Immunization With 1 or 2 Doses of Inactivated Poliovirus Vaccine: Systematic Review and Meta-analysis — Immunogenicity and Effectiveness of Routine Immunization With 1 or 2 Doses of Inactivated Poliovirus Vaccine: Systematic Review and Meta-analysis — Supplementary Data 

# Immunogenicity and Effectiveness of Routine Immunization With 1 or 2 Doses of Inactivated Poliovirus Vaccine: Systematic Review and Meta-analysis

## Supplementary Data

Supplementary Data

**Files in this Data Supplement:**

- Supplementary Data - Docx file
- Supplementary Figure 1 - docx file
- Supplementary Table 1 - docx file
- Supplementary Table 2 - docx file
