# Supplementary material for: Immunogenicity and Effectiveness of Routine Immunization With 1 or 2 Doses of Inactivated Poliovirus Vaccine: Systematic Review and Meta-analysis
Source: J Infect Dis. 2014 Nov 1;210(Suppl 1):S439–46. doi: 10.1093/infdis/jit601 (PMC4197908; doi:10.1093/infdis/jit601)
Supplement: Supplementary Data [file supp_jit601_jit601supp.docx]

***Supplementary Methods***

*Systematic review protocol*

Data on seroconversion after 1 or 2 full or fractional doses of enhanced potency IPV was extracted from published studies. Potentially relevant articles were identified through a search of the Web of Knowledge^SM^ collection of databases (including Web of Science and Medline; Thomson Reuters) on the 18^th^ June 2013 using the search term ((polio* and vaccin* and (antibod* or seroconver* or sero-conver*) and (inactiv* or killed or salk))) in the title, abstract or keywords. Articles were screened for relevance by reading the title and abstract, and those that contained, or potentially contained, data on the immunogenicity of enhanced potency IPV after fewer than 3 doses as part of a primary immunisation series of healthy infants were examined in full-text. After reading in full-text, articles were retained if they reported on seroconversion in a neutralization assay following 1 or 2 doses of enhanced potency IPV, administered intradermally (ID) or intramuscularly (IM), as primary immunisation in healthy humans, alone or in a combination product. Articles were excluded if they only reported on booster vaccination; used older, lower potency IPV (compared with modern ‘enhanced potency’ IPV [[1](#_ENREF_1)]); included fewer than 10 individuals; provided insufficient information to determine the proportion of vaccine recipients undergoing seroconversion; or duplicated results reported in an earlier publication. Articles read in full-text were also examined for citations to additional studies fulfilling the inclusion and exclusion criteria that were not returned by the initial database search.

For each article included in the systematic review the numbers of children examined and the number undergoing seroconversion were entered by the author into Excel. The proportion undergoing seroconversion was calculated and compared with the original report to check for any typographical errors. Information entered into Excel also included the age at administration of the vaccine dose(s), the age at determination of seroconversion, the definition of seroconversion, the route of administration of vaccine, the device used for administration, the product used (stand-alone or combined), vaccine antigen content, the geometric mean titre of neutralising antibodies (when reported) and study location. The potential for secondary exposure to oral poliovirus vaccine, which could inflate the proportion of children undergoing seroconversion, was assessed for each study based on the study date, location and any other information in the full-text article.

*Statistical analysis*

Exact binomial confidence intervals were calculated for each study using the F-distribution. Heterogeneity in the proportion sero-converting across studies was assessed using the χ^2^-test. Trends in the expected proportion undergoing seroconversion with age at administration of the first dose of IPV were assessed on the basis of the maximum likelihood fit of a linear or exponential relationship. Formal tests for the association between seroconversion after 1 or 2 doses of IPV and antigen content/route of administration of the vaccine, age at administration and interval between doses (for 2 doses only) were performed for each serotype using a mixed effects binomial regression that included a normally-distributed random intercept for each published study to account for within-study correlation. Statistical analyses were performed in Excel and in the R programming language using the lme4 package [[2](#_ENREF_2)].

*References*

1. Mellander L, Bottiger M, Hanson LA, Taranger J, Carlsson B. Avidity and titers of the antibody-response to 2 inactivated poliovirus vaccines with different antigen content. Acta Paediatrica **1983**; 82:552-556.

2. R Development Core Team. R: A language and environment for statistical computing: R Foundation for Statistical Computing, Vienna, Austria. ISBN 3-900051-07-0, **2013**.
