# Supplementary material for: Immunogenicity and Effectiveness of Routine Immunization With 1 or 2 Doses of Inactivated Poliovirus Vaccine: Systematic Review and Meta-analysis
Source: J Infect Dis. 2014 Nov 1;210(Suppl 1):S439–46. doi: 10.1093/infdis/jit601 (PMC4197908; doi:10.1093/infdis/jit601)
Supplement: Supplementary Data [file supp_jit601_jit601supp_fig1.docx]

**Figure S1** **Flow chart showing the number of studies examined as part of the systematic review** **according to PRISMA guidelines**

Records identified through database searching

Web of Knowledge

(n = 958)

Additional records identified through literature cited

(n = 0)

Records screened
(n = 958)

Records excluded after screening titles and abstracts
(n = 881)

Full-text articles assessed for eligibility
(n = 77)

Articles that did not meet the inclusion criteria

(n = 19)

Articles that met the inclusion criteria
(n = 58)

Full-text articles excluded
(n = 46)

- booster vaccination (n = 2)
- old IPV (n = 3)
- < 10 individuals (n = 1)
- duplicates (n = 7)
- insufficient information (e.g reported seroprevalence or geometric mean titres only) (n = 33)

Articles meeting all criteria

(n = 12 articles describing 20 independent study arms)
