# Supplementary material for: Immunogenicity and Effectiveness of Routine Immunization With 1 or 2 Doses of Inactivated Poliovirus Vaccine: Systematic Review and Meta-analysis
Source: J Infect Dis. 2014 Nov 1;210(Suppl 1):S439–46. doi: 10.1093/infdis/jit601 (PMC4197908; doi:10.1093/infdis/jit601)
Supplement: Supplementary Data [file supp_jit601_jit601supp_table1.docx]

**Table S1 Seroconversion after 1 full or fractional (1/5) dose of inactivated poliovirus vaccine**

|  | Study | Location | Age at administration | Age at which seroconversion measured | Definition of sero-conversion | Seroconversion by serotype (%) | | | Notes |
| --- | --- | --- | --- | --- | --- | --- | --- | --- | --- |
|  |  |  |  |  |  | 1 | 2 | 3 |  |
| ***Full dose*** | |  |  |  |  |  |  |  |  |
|  | Resik et al. 2010 [[1](#_ENREF_1)] | Cuba | 6 weeks | 10 weeks | A | 19 (34/177) | 36 (63/177) | 42 (75/177) |  |
|  | Mohammed et al. 2010 [[2](#_ENREF_2)] | Oman | 2 months | 4 months | A | 22 (40/182) | 32 (57/180) | 45 (82/183) |  |
|  | Jain et al. 1997 [[3](#_ENREF_3)] | India | birth | 6 weeks | B | 48 (24/50) | 64 (32/50) | 30 (15/50) |  |
|  | Linder et al. 1995 [[4](#_ENREF_4)] | Israel | 5-10 days | 1 month | A | 8 (3/39) | 15 (6/39) | 28 (11/39) |  |
|  | Resik et al 2013 [[5](#_ENREF_5)] | Cuba | 4 months | 8 months | A | 46 (71/153) | 63 (96/153) | 32 (49/153) |  |
|  | Simasathien et al 1994 [[6](#_ENREF_6)] | Thailand | 2 months | 3 months | B | 25 (26/103) | 39 (40/103) | 28 (29/103) |  |
|  | McBean et al. 1988 [[7](#_ENREF_7)]* | USA | 2 months | 4 months | A | 42 (130/309) | 35 (109/311) | 54 (165/306) |  |
|  | McBean et al. 1988 [[7](#_ENREF_7)]* | USA | 2 months | 4 months | A | 35 (109/312) | 43 (139/324) | 61 (190/311) |  |
|  | *Overall* |  |  |  |  | *33 (437/1325)* | *41 (542/1337)* | *47 (616/1322)* | |
| ***Fractional dose*** | |  |  |  |  |  |  |  |  |
|  | Resik et al 2010 [[1](#_ENREF_1)] | Cuba | 6 weeks | 10 weeks | A | 5 (9/187) | 19 (35/187) | 7 (14/187) | Biojector 2000 |
|  | Mohammed et al. 2010 [[2](#_ENREF_2)] | Oman | 2 months | 4 months | A | 10 (19/184) | 17 (31/185) | 9 (17/186) | Biojector 2000 |
|  | Resik et al 2013 [[5](#_ENREF_5)] | Cuba | 4 months | 8 months | A | 17 (26/157) | 47 (74/157) | 15 (23/157) | Biojector 2000 |
|  | *Overall* |  |  |  |  | *10 (54/528)* | *26 (140/529)* | *10 (54/530)* |  |

Definitions of seroconversion, A: 4-fold increase in serum neutralising antibodies over expected titre based on 28-30 day half-life of maternal antibodies, or a change from undetectable to detectable antibodies; B: 4-fold increase over baseline or change from undetectable to detectable antibodies. *In the study by McBean et al. (1988) vaccine from 2 different manufacturers was assessed and are therefore reported separately, the first listed above contained 24 to 38, 4 to 7 and 28 to 36 D-antigen units for types 1, 2 and 3 respectively, and the second 20 to 25, 7 to 9 and 26 to 30 respectively.

**Study references**

1. Resik S, Tejeda A, Lago PM, Diaz M, Carmenates A, et al. (2010) Randomized controlled clinical trial of fractional doses of inactivated poliovirus vaccine administered intradermally by needle-free device in Cuba. J Infect Dis 201: 1344-1352.

2. Mohammed AJ, AlAwaidy S, Bawikar S, Kurup PJ, Elamir E, et al. (2010) Fractional doses of inactivated poliovirus vaccine in Oman. N Engl J Med 362: 2351-2359.

3. Jain PK, Dutta AK, Nangia S, Khare S, Saili A (1997) Seroconversion following killed polio vaccine in neonates. Indian J Pediatr 64: 511-515.

4. Linder N, Yaron M, Handsher R, Kuint J, Birenbaum E, et al. (1995) Early immunization with inactivated poliovirus vaccine in premature infants. The Journal of pediatrics 127: 128-130.

5. Resik S, Tejeda A, Sutter RW, Diaz M, Sarmiento L, et al. (2013) Priming after a fractional dose of inactivated poliovirus vaccine. New Engl J Med 368: 416-424.

6. Simasathien S, Migasena S, Beuvery C, Vansteenis G, Samakoses R, et al. (1994) Comparison of enhanced potency inactivated poliovirus vaccine (eIPV) versus standard oral poliovirus vaccine (OPV) in thai infants. Scand J Infect Dis 26: 731-738.

7. McBean AM, Thoms ML, Albrecht P, Cuthie JC, Bernier R (1988) Serologic response to oral polio vaccine and enhanced-potency inactivated polio vaccines. Am J Epidemiol 128: 615-628.
