# Supplementary material for: Immunogenicity and Effectiveness of Routine Immunization With 1 or 2 Doses of Inactivated Poliovirus Vaccine: Systematic Review and Meta-analysis
Source: J Infect Dis. 2014 Nov 1;210(Suppl 1):S439–46. doi: 10.1093/infdis/jit601 (PMC4197908; doi:10.1093/infdis/jit601)
Supplement: Supplementary Data [file supp_jit601_jit601supp_table2.docx]

**Table S2 Seroconversion after 2 full or fractional (1/5) doses of inactivated poliovirus vaccine**

|  | Study | Location | Schedule (age at doses) | Interval between doses (weeks) | Age at which seroconversion measured | Definition of seroconversion | Seroconversion (%) | | | notes |
| --- | --- | --- | --- | --- | --- | --- | --- | --- | --- | --- |
|  |  |  |  |  |  |  | 1 | 2 | 3 |  |
| ***Full dose*** | |  |  |  |  |  |  |  |  |  |
|  | Resik et al. 2010 [[1](#_ENREF_1)] | Cuba | 6, 10 weeks | 4 | 14 weeks | A | 63 (112/177) | 76 (134/177) | 93 (165/177) | ++ |
|  | Mohammed et al. 2010 [[2](#_ENREF_2)] | Oman | 2, 4 months | 9 | 6 months | A | 91 (165/182) | 91 (163/180) | 96 (175/183) | ++ |
|  | Jain et al. 1997 [[3](#_ENREF_3)] | India | birth, 6 weeks | 6 | 10 weeks | B | 80 (20/25) | 68 (17/25) | 76 (19/25) |  |
|  | Linder et al. 1995 [[4](#_ENREF_4)] | Israel | 5-10, 60 days | 8 | 3 months | A | 28 (9/32) | 41 (13/32) | 56 (18/32) |  |
|  | Resik et al 2013 [[5](#_ENREF_5)] | Cuba | 4, 8 months | 17 | 9 months | A | 100 (153/153) | 100 (153/153) | 99 (152/153) |  |
|  | Singh et al 1992 [[6](#_ENREF_6)] | India | 6-7, 10-11 or 14-15 weeks | NA | 4-6 weeks later | A | 94 (30/32) | 91 (29/32) | 84 (27/32) |  |
|  | Singh et al 1992 [[6](#_ENREF_6)] | India | 8-24, 12-28 or 16-21 weeks | NA | 4-6 weeks later | A | 90 (18/20) | 90 (18/20) | 95 (19/20) |  |
|  | WHO Collab 1997 [[7](#_ENREF_7)] | Oman | 6, 10 weeks | 4 | 14 weeks | A | 71 (96/136) | 83 (113/136) | 81 (110/136) | combined DPT-IPV |
|  | WHO Collab 1997 [[7](#_ENREF_7)] | Thailand | 6, 10 weeks | 4 | 14 weeks | A | 40 (56/141) | 48 (67/141) | 79 (111/141) | combined DPT-IPV |
|  | IPV Study Grp 2007 [[8](#_ENREF_8)] | Cuba | 8, 16 weeks | 8 | 4 weeks later | A | 90 (65/72) | 89 (64/72) | 90 (65/72) | combined DPT-IPV |
|  | Simoes et al. 1985 [[9](#_ENREF_9)] | India | 6-7, 10-11 weeks | 4 | 4 weeks later | A | 95 (61/64) | 75 (48/64) | 97 (62/64) | combined DPT-IPV |
|  | Simoes et al. 1985 [[9](#_ENREF_9)] | India | 6-7, 14-15 weeks | 8 | 4 weeks later | A | 95 (71/75) | 83 (62/75) | 96 (72/75) | combined DPT-IPV |
|  | Simoes et al. 1985 [[9](#_ENREF_9)] | India | 8-12, 12-16 weeks | 4 | 4 weeks later | A | 94 (16/17) | 88 (15/17) | 100 (17/17) | combined DPT-IPV |
|  | Simoes et al. 1985 [[9](#_ENREF_9)] | India | 8-12, 16-20 weeks | 8 | 4 weeks later | A | 100 (21/21) | 95 (20/21) | 100 (21/21) | combined DPT-IPV |
|  | Simoes et al. 1985 [[9](#_ENREF_9)] | India | 13-45, 17-49 weeks | 4 | 4 weeks later | A | 100 (19/19) | 89 (17/19) | 89 (17/19) | combined DPT-IPV |
|  | Simoes et al. 1985 [[9](#_ENREF_9)] | India | 13-45, 21-53 weeks | 8 | 4 weeks later | A | 100 (18/18) | 100 (18/18) | 100 (18/18) | combined DPT-IPV |
|  | *Overall* |  |  |  |  |  | *79 (930/1184)* | *80 (951/1182)* | *90 (1068/1185)* | |
| ***Fractional dose*** | |  |  |  |  |  |  |  |  |  |
|  | Resik et al 2010 [[1](#_ENREF_1)] | Cuba | 6, 10 weeks | 4 | 14 weeks | A | 27 (51/187) | 55 (103/187) | 43 (81/187) | Biojector 2000, ++ |
|  | Mohammed et al. 2010 [[2](#_ENREF_2)] | Oman | 2, 4 months | 9 | 6 months | A | 70 (129/184) | 72 (134/185) | 72 (134/186) | Biojector 2000, ++ |
|  | Resik et al 2013 [[5](#_ENREF_5)] | Cuba | 4, 8 months | 17 | 9 months | A | 94 (147/157) | 98 (154/157) | 93 (146/157) | Biojector 2000 |
|  | Nirmal et al. 1998 [[10](#_ENREF_10)] | India | 6, 14 weeks | 8 | 18 weeks | A | 90 (27/30) | 70 (21/30) | 97 (29/30) | needle and syringe |
|  | *Overall* |  |  |  |  |  | *63 (354/558)* | *74 (412/559)* | *70 (390/560)* |  |

Seroconversion definitions as for Table S1. ++ cumulative seroconversion reported, which may exclude a small number meeting criterion for seroconversion following both doses but not for either dose 1 or 2

**Study references**

1. Resik S, Tejeda A, Lago PM, Diaz M, Carmenates A, et al. (2010) Randomized controlled clinical trial of fractional doses of inactivated poliovirus vaccine administered intradermally by needle-free device in Cuba. J Infect Dis 201: 1344-1352.

2. Mohammed AJ, AlAwaidy S, Bawikar S, Kurup PJ, Elamir E, et al. (2010) Fractional doses of inactivated poliovirus vaccine in Oman. N Engl J Med 362: 2351-2359.

3. Jain PK, Dutta AK, Nangia S, Khare S, Saili A (1997) Seroconversion following killed polio vaccine in neonates. Indian J Pediatr 64: 511-515.

4. Linder N, Yaron M, Handsher R, Kuint J, Birenbaum E, et al. (1995) Early immunization with inactivated poliovirus vaccine in premature infants. The Journal of pediatrics 127: 128-130.

5. Resik S, Tejeda A, Sutter RW, Diaz M, Sarmiento L, et al. (2013) Priming after a fractional dose of inactivated poliovirus vaccine. New Engl J Med 368: 416-424.

6. Singh J, Ravi RN, Dutta AK, Kumari S, Khare S (1992) Immunogenicity of enhanced potency inactivated polio vaccine. Indian pediatrics 29: 1353-1356.

7. WHO Collaborative Study Group on Oral and Inactivated Poliovirus Vaccines (1997) Combined immunization of infants with oral and inactivated poliovirus vaccines: results of a randomized trial in The Gambia, Oman, and Thailand. J Infect Dis 175 (suppl. 1): S215-227.

8. Galindo M, Lago PM, Caceres V, Landaverde M, Sutter R. (Cuba IPV Study Collaborative Group) (2007) Randomized, placebo-controlled trial of inactivated poliovirus vaccine in Cuba. New Engl J Med 356: 1536-1544.

9. Simoes EAF, Padmini B, Steinhoff MC, Jadhav M, John TJ (1985) Antibody-response of infants to 2 doses of inactivated poliovirus vaccine of enhanced potency. Am J Dis Child 139: 977-980.

10. Nirmal S, Cherian T, Samuel BU, Rajasingh J, Raghupathy P, et al. (1998) Immune response of infants to fractional doses of intradermally administered inactivated poliovirus vaccine. Vaccine 16: 928-931.
